# EEG in game user analysis: A framework for expertise classification during gameplay

**DOI:** 10.1371/journal.pone.0246913

**Published:** 2021-06-18

**Authors:** Tehmina Hafeez, Sanay Muhammad Umar Saeed, Aamir Arsalan, Syed Muhammad Anwar, Muhammad Usman Ashraf, Khalid Alsubhi

**Affiliations:** 1 Department of Computer Engineering, University of Engineering and Technology, Taxila, Pakistan; 2 Department of Software Engineering, University of Engineering and Technology, Taxila, Pakistan; 3 Department of Computer Science, University of Management and Technology, Lahore (Sialkot), Pakistan; 4 Department of Computer Science, King Abdul Aziz University, Jeddah, Saudi Arabia; Universidad de Granada, SPAIN

## Abstract

Video games have become a ubiquitous part of demographically diverse cultures. Numerous studies have focused on analyzing the cognitive aspects involved in game playing that could help in providing an optimal gaming experience by improving video game design. To this end, we present a framework for classifying the game player’s expertise level using wearable electroencephalography (EEG) headset. We hypothesize that expert and novice players’ brain activity is different, which can be classified using frequency domain features extracted from EEG signals of the game player. A systematic channel reduction approach is presented using a correlation-based attribute evaluation method. This approach lead us in identifying two significant EEG channels, i.e., AF3 and P7, among fourteen channels available in Emotiv EPOC headset. In particular, features extracted from these two EEG channels contributed the most to the video game player’s expertise level classification. This finding is validated by performing statistical analysis (t-test) over the extracted features. Moreover, among multiple classifiers used, K-nearest neighbor is the best classifier in classifying game player’s expertise level with a classification accuracy of up to 98.04% (without data balancing) and 98.33% (with data balancing).

## Introduction

Technology advancements have revolutionized the gaming industry leading to a dynamic shift from computer games to mobile games. This shift has increased the number of active mobile game players to around 2.1 billion, making the gaming industry one of the highest profit earning entertainment industries. In a significant milestone, the video game industry contributed 80% of the $36 billion profit earned from all software-related industries in the year 2018 in United States alone [1]. According to a global games market report, it is expected that the gaming market segment will be worth $180.1 billion by the year 2021 [2]. This rapid growth in the gaming industry creates significant competition for game developers to capture attention of a diverse population of video game players. A major factor in game development deals with improving the user experience, which demands an accurate cognitive assessment of game players to improve user interaction and enhance the gaming experience [3, 4]. Towards this, our aim is to develop a method that uses wearable sensors, in particular electroencephalography (EEG), for game user analysis. The expert-novice paradigm is an important consideration in game user analysis. This could be used for better game design, where gameplay is adaptive to the player responses. This would in turn make games more engaging for the end user. Game design can also benefit by analyzing various sub-parts of the game and how a player responds to these sub-segments. Expert-novice paradigm for mobile game players is a developing field, where the recent wide-spread availability of wearable sensors could lead towards significant progress in the near future.

The term *player experience*, in the context of game user research (GUR), is derived from the discipline of human-computer interaction. In particular, player experience is formally explored using constructs such as flow, immersion, challenge-skill balance, affect, presence, motivation, and tension [5]. The player experience is commonly measured by self-reported questionnaires [6], which includes immersive experience questionnaire (IEQ) [7], game experience questionnaire (GEQ), in-game GEQ (iGEQ) [8], and game engagement questionnaire (GEnQ) [9, 10]. There are a large number of questionnaires currently in use, yet the convergence in their usability is minimal. This introduces several challenges both in terms of selecting suitable questionnaires and comparing results across different studies. A hybrid approach can be a possible solution to some of the known challenges [11]. Among various questionnaires used for player experience analysis, none of them are aimed towards identifying the expertise level of the game player. Subjective evaluation using self-labeling is widely used for this purpose, which is gradually replaced by objective evaluation methods. Objective evaluation methods perform a comprehensive analysis of the physiological observations recorded from game players during the period of gameplay. Such observations are efficient in analyzing the cognitive and behavioral aspects of the game player. Since the last decade, the cognitive and behavioral analysis of the game player is becoming an emerging research topic in the GUR. [12–14].

Physiological measures such as electrocardiography (ECG) [15], electromyography (EMG) [16], electrodermal activity (EDA) [15, 17], and electroencephalography, are significantly gaining importance in GUR [18–21]. In particular, games that provide adaptive features in response to brain signals are known as *neuro-feedback games*. The usability of physiological measures in the game design of neuro-feedback games has been demonstrated using well-designed experiments [22]. Neuro-feedback games related to EEG can be grouped into three categories including, 1) active (for instance, brain arena, brain runner, and shooter games), 2) reactive (for instance, checker game, memory game, and space invaders), and 3) passive (for instance, matrix game, brain ball, and mind ninja games) [23]. Contrary to conventional game control, a neuro-feedback game use brain signals to adapt the game environment, i.e., either control the game speed or adapt the game design according to cognitive and motor skills of the game player [24]. EEG-based neuro-feedback is increasingly used for serious games. For instance, *mindlight* is a serious neuro-feedback game that is developed to reduce anxiety in children [25]. Similarly, 2D and 3D EEG-based games such as *brain chi*, *dancing robot*, *pipe*, and *escape* are successfully deployed for concentration training purposes [26]. Recently, a multiplayer car racing game was proposed to improve attention level of a user [27]. In particular, it was shown that varying game difficulty in correspondence with the variation in the emotions of the game player helps in preserving their interest and maintains player engagement. It was reported that both healthy and physically impaired people find adaptive neuro-feedback games as motivating, attractive, and more challenging than non-adaptive games [13]. Furthermore, such responsive games are found to be successful for attention enhancement and better cognition in game players [28].

Herein, we propose a method for classification of expertise level of the game player. Our proposed framework would use features extracted from the EEG signals observed during the game-play. The proposed EEG-based game player expertise classification framework can be augmented with an adaptive neuro-feedback game design. It will allow adapting the game difficulty according to the variations in the brain activity of the game player. Thus, it will help in maintaining the skill-competence, attention, and cognition of the game player. This is a step towards improving the player experience by assimilating the concepts of dynamic difficulty adjustment (DDA) and neuro-feedback in game design.

### Related work

Electrophysiological methods provide an objective, continuous, and impartial measure for analyzing the player experience [29]. EEG has significantly gained importance in GUR and is shown to be an effective tool in game studies [20]. For instance, EEG was found to be useful in differentiating stressed and relaxed states of the game player during the racing and first-person shooter games [21]. Moreover, the level of motivation and relaxation in the game players was investigated using the EEG data [29, 30]. Attention differences among video game players and non-video game players were also investigated using EEG signals; by recording the steady-state visual evoked potential and event-related potentials [31]. The correlation of EEG frequency bands with valence, arousal, and engagement was investigated to find the best classifier for various cognitive-affective states of the game player [32]. Wearable EEG devices were found to be a discreet, reliable, and portable way for observing and discriminating the cognitive-affective states of the game player [33]. In particular, the Emotiv EPOC headset is a low-cost and widely used device in the game studies. It was reported that Emotiv EPOC could be a better substitute than laboratory-based EEG systems for analyzing the game player experience [34].

While expertise level classification is important, there are limited studies available in the literature [35–37]. We argue that for GUR, this area is significant and hence needs a more systematic analysis. It has been observed that a real-time analysis of game player experience helps in keeping the player positively influenced, motivated, immersed, engaged, relaxed, and improve the cognitive abilities [38–40]. In [35], researchers found that the player’s expertise and competency can be better classified using features from the EEG signals and regression-based ridge estimator classifier. In [36, 41], mobile game players’ expertise was classified using EEG signals and machine learning algorithms, where the Naive Bayes (NB) was found to be better among other classifiers used. It is observed that the reported classification accuracy needs to be improved further to develop a better game design based on the expertise level of the game player. Herein, we aim to achieve an improved and accurate classification of the expertise level (expert/novice) of the game player.

### Our contributions

We aim to classify the game player’s expertise level in one of the two categories, i.e., expert or novice, by utilizing features of the most relevant EEG channels. Hence the computational overhead for analysis and real-time implementation of such a system will reduce. Further, we intend to improve the classification accuracy of the game player’s expertise level. Towards this, we utilize well-established features and classification algorithms. However, we intend to identify EEG electrodes which are more suited for the task at hand using a wearable device. Henceforth, this study aims to contribute in the following ways in GUR,

1. An improvement in the classification accuracy of the player expertise level during gameplay using features extracted from EEG signals acquired using a wearable device.
2. A computationally efficient mechanism for the classification of game player expertise level by effective EEG channel selection and feature-length reduction.

The rest of the paper is organized as follows. pm describes the proposed methodology along with the materials and methods used in the study, er presents the experimental results followed by the discussion and conclusion in disc and conc, respectively.

## Proposed methodology

**Fig 1:**
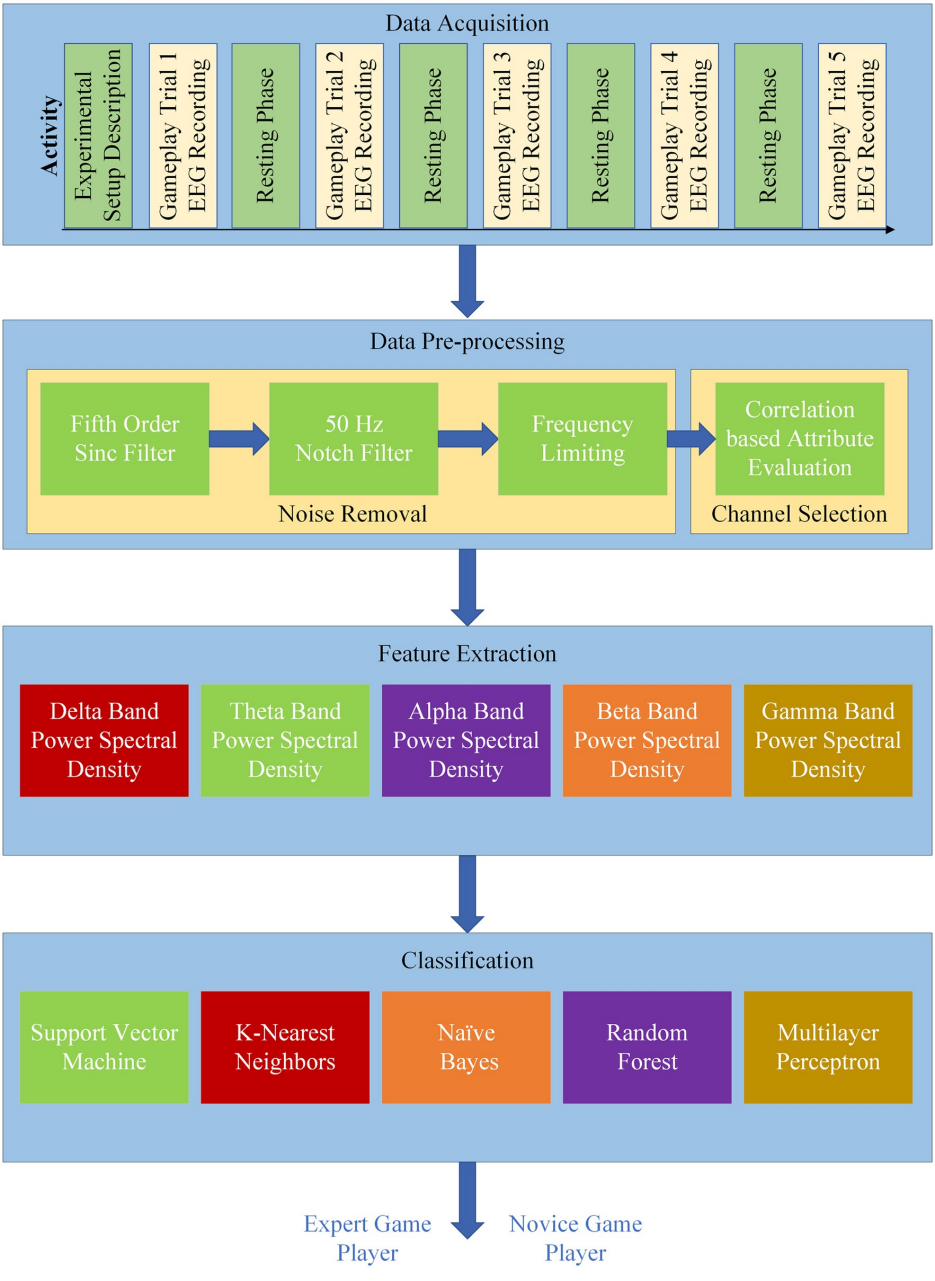
Block diagram of the proposed methodology for the expert-novice classification of the game player.

A block diagram of the proposed scheme for EEG-based game player expertise classification is presented in Fig 1. It consists of three main stages, namely 1) data processing including pre-processing and channel selection, 2) feature extraction, and 3) expertise classification. The EEG data used in this study were collected in a previous study [41] and is publicly available at https://www.kaggle.com/enversyed/game-player-expertise-classification. However, a brief overview of the EEG data and experimental procedure for EEG data acquisition is described here for completeness. The following subsections present details for each of the blocks used in the proposed framework (Fig 1).

### Data acquisition

Ten healthy subjects (one female and nine males), with an average age of 20.37 years, voluntarily participated in the experimental study. All participants belonged to the Asia-Pacific region, having the same educational background, and reported experience in playing games. A popular android based game, *Temple Run*, was used as a stimulus in this experimental study for EEG data acquisition. It is an endless game, which continues until either a demonic monkey eats the character or the character falls in the river or is hit by an obstacle. The experimental protocol followed was designed following the recommendations of the Helsinki declaration.

**Fig 2:**
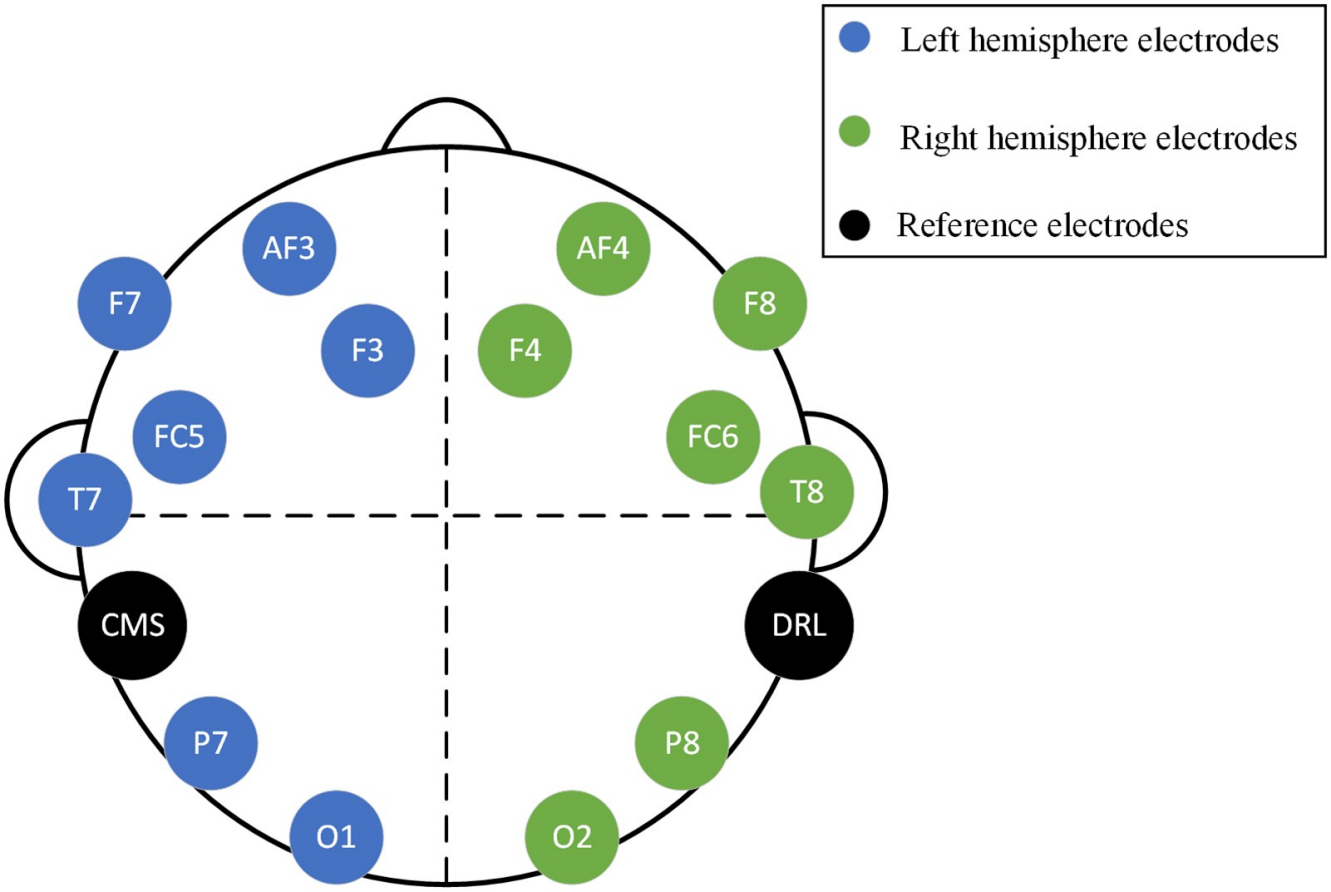
10-20 electrode placing system for placing the Emotiv EPOC headset [41].

EEG data acquisition was performed using a 14-channel Emotiv EPOC headset by placing it over the participant’s scalp. Emotiv EPOC is a flexible, high resolution, multi-channel wireless headset and provides fourteen electrodes arranged according to the 10-20 electrode placing system (as shown in Fig 2). These electrodes include AF3, AF4, F7, F8, F3, F4, FC5, FC6, T7, T8, O1, O2, P7, and P8. Further, two additional reference electrodes (CMS and DRL) are positioned on the rear of both ears. The headset provides good temporal resolution and electrodes sufficiently cover the scalp to capture the brain’s electrical activity. For instance, electrodes AF3 and AF4 cover the pre-frontal region, F3, F4, F7, and F8 cover the frontal region, FC5 and FC6 cover the frontal-central region, T7 and T8 cover the temporal region, P7 and P8 cover the parietal region, and O1 and O2 capture the neural activity from the occipital region of the brain. While the signal quality from such wearable devices could be questionable for clinical studies, it has been shown that the acquired data yields significant results in less critical applications.

During the experiment, EEG electrodes were soaked with a saline liquid to acquire good quality signals. The study was approved by the board of postgraduate studies, Department of Computer engineering, UET Taxila. All participants were informed about the experimental procedure, and written consent was obtained. It was followed by filling the demographic sheet comprising information including name, age, education, and gender. The participants were provided with a comfortable chair and a smartphone with a pre-installed Temple Run game. The room chosen for EEG data acquisition was acoustically noise-free. Moreover, the electric cabling was avoided near the experimental setup to keep the environmental noise to a bare minimum. Additionally, proper synchronization was done between the Emotiv EPOC headset and Emotiv software development kit (SDK) to avoid erroneous or faulty data acquisition.

**Fig 3:**
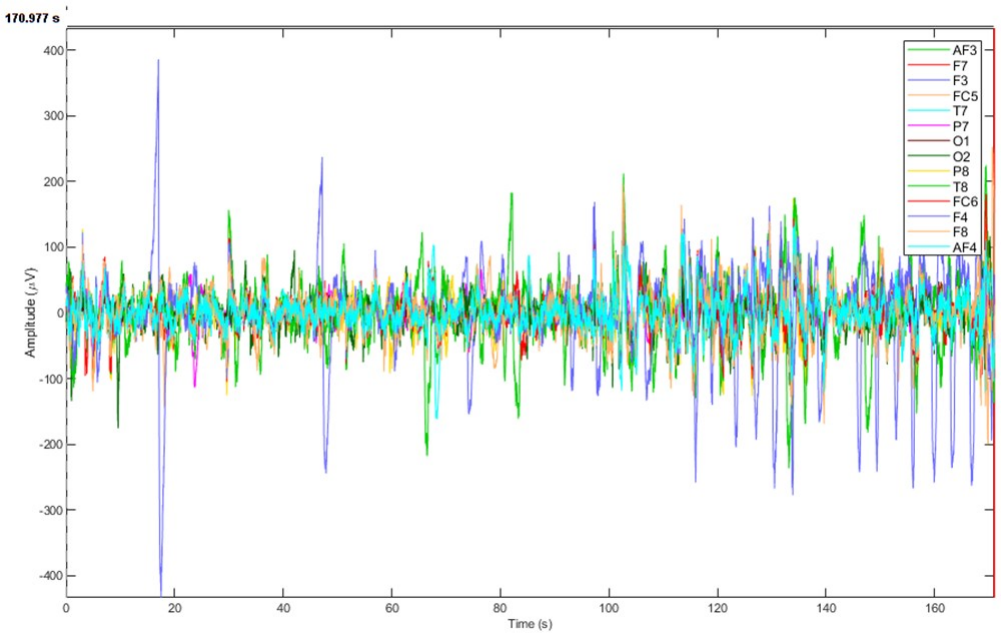
A plot showing raw data acquired from Emotiv EPOC Headset.

EEG data of each participant was recorded for five trials of gameplay. Each trial of the gameplay was separated by a resting phase of 60 seconds. The average gameplay time observed for each participant was 24.7 minutes. EEG data were recorded at a sampling rate of 128*Hz* and was saved in the European data format (EDF) using Emotiv SDK. The recorded data was transferred via Bluetooth to a computer for further offline analysis. Fig 3 Game score for each participant after each trial was recorded, and an average score was calculated for all of the participant’s five trials. A threshold (*T*) on the game score of each participant was calculated by adding the scores of all trials of all participants and dividing it by the total number of participants [36]. The threshold (*T*) was calculated as follows,

$$\begin{array}{c} T=\frac{\sum_{m}^{p}\sum_{n}^{t}S\left(m,n\right)}{p\times t}, \end{array}$$ (1)

where *p* is the total number of participants, *t* is the total number of trials, and *S(m,n)* is the score of *n*^*th*^ trial of the *m*^*th*^ participant. The participant with an average game score above this threshold was labeled as an expert, while those with lower scores were labeled as novice. These assigned labels also corresponded to the subject’s self-reported expertise. Thus, four participants were identified as an expert, while six participants were identified as novice based on the threshold value.

### Data pre-processing

The data pre-processing stage consists of two sub-stages, i.e., pre-processing for noise removal and channel selection phase for the selection of the most significant EEG channels. Hence, we come up with relevant EEG channels whose features contributed the most in classifying game player expertise level. The details are presented in the following subsections.

#### Noise removal

EEG signals have micro-volt amplitude making them highly prone to contamination by various artifacts. These artifacts, including muscle movement, electrical lines, and electrode movement should be carefully removed prior to further processing of the EEG data. Artifacts such as eye blinks were automatically detected and removed by Emotiv SDK. EEG Emotiv Epoc system uses the distribution of sensors around the face to triangulate muscle sources and to build classification systems to identify specific muscle signals. It characterizes the kind of filtering that is needed to remove EMG from the data. Emotiv EPOC has a built-in 5^*th*^ order sinc filter, which acts as a low pass filter with a low cut off frequency and higher attenuation in the stop-band to reduce noise in EEG signals. It also has notch filters at 50 and 60 Hz, which acts as a band-stop filter to rectify noise generated by power supply lines.

#### Channel selection

After the pre-processing step, the EEG signals were subjected to channel selection, where the average power of time-domain EEG signals was computed across all fourteen channels. Mathematically average power was calculated as follows

$$\begin{array}{c} Power_{avg}=\frac{1}{N}\sum_{n=0}^{N-1}{\left|x[n]\right|}^{2}, \end{array}$$ (2)

where *x* is the EEG signal with length *N* across each EEG channel. After time-domain power computation, *correlation-based feature selection* was applied to select the most relevant channels for game player’s expertise classification [42]. *Correlation-based attribute selection* is based on the principle that an attribute having a high correlation with the class labels must be selected, whereas attributes which have a high correlation with other attributes should be ignored. The correlation between each attribute and output class was mathematically calculated as follows,

$$\begin{array}{c} c=\frac{\left(M\sum FO-(\sum F)(\sum O)\right)}{\sqrt{\left[M\sum F^{2}\right)-{\left(\sum F\right)}^{2}]\left[M\sum O^{2}-{\left(\sum O\right)}^{2}\right]}}, \end{array}$$ (3)

where *M* is the total number of pair of scores, *F* is the feature vector, *O* is the output class, *c* is the *Pearson’s correlation coefficient*, ∑*F* is the sum of *F* scores, ∑*FO* is the sum of products of paired scores, ∑*F*^2^ is the squared *F* scores, ∑*O* is the sum of output class and ∑*O*^2^ is the sum of squared *O* scores.

The correlation-based feature selection was applied in *Weka* tool using the *Correlation AttributeEval* method, which evaluates the correlation of all fourteen EEG channels with the output class and presents correlation coefficients in a ranked order. Out of the fourteen EEG channels, the top-ranked channels (AF3, O1, P7 and T7) were chosen, which were highly correlated with the output class to reduce the computational overhead and avoid the chances of over-fitting. Further, frequency domain features from these selected channels were extracted for game player’s expertise classification.

### Feature extraction

In general, *power spectral density (PSD)* represents the power distribution of a signal over a particular frequency. In this study, the analysis of the recorded EEG data was performed in the frequency domain by computing the PSD of the time domain signal. To this end, *Welch* method was employed using *Brainstorm3* toolbox in MATLAB. The *Welch* method was used to compute the power spectrum of the input signal by dividing it into 50% overlapping sequences of the specified length followed by the application of the *Hamming* window to each of the overlapped sequences. Further, *Fast Fourier Transform (FFT)* of each sub-region was computed. Finally, power from the FFT coefficients of all overlapped windows was averaged. Thus, the time-domain EEG signal was represented by the power spectrum of its frequency bands [43]. The extracted PSD features from each channel included EEG frequency bands in the following ranges: *delta (2-4 Hz), theta (5-7 Hz), alpha (8-12 Hz), beta (13-29 Hz), and gamma (30-45 Hz)*. These features were extracted for those EEG channels which resulted in a high correlation with the output class and hence were selected as discussed in Channel Selection section. It has been shown that these frequency bands have significance in various human activities [44, 45]. For instance, higher theta values corresponded to focused attention and were found to be a differentiating factor between expert and novice shooters in an exhibition match [46]. Moreover, it was shown that alpha band activity reflects task-specific demands in parieto-occipital regions, therefore, novices exhibit more cortical activation as compared to experts. It was shown that theta/beta ratio provided useful information in the study of affective and emotional regulation [35]. In another study, delta and gamma bands were found useful in pleasantness classification [47].

### Game player expertise classification

For the expert-novice classification of game player, five supervised machine learning algorithms were used, including support vector machine, the Naïve Bayes (NB), K-nearest neighbor (KNN), multiplayer perceptron (MLP), and random forest (RF). The details of these algorithms are presented in the following text for completeness.

#### Support vector machine (SVM)

SVM is a supervised machine learning algorithm used for both classification and regression problems [48]. It uses linear, non-linear, Gaussian, or polynomial kernels to achieve a hyperplane that efficiently separates the input data. Herein, we classified the input feature set in two classes, i.e., expert and novice, using the output class labels and used a linear kernel. In a previous study [41], game player expertise was classified with 80% accuracy using an SVM classifier. In another study, SVM had classified the game player’s expertise with 82% and 86% classification accuracy when features of 14 and 4 EEG channels were utilized respectively [36]. Apart from game player expertise classification, SVM is well suited for the classification of emotions in general gameplay events [32]. As observed in the previous studies, the efficiency of the SVM algorithm provided a rationale for using it in the current study for classifying the expertise level of a game player.

#### Naive Bayes (NB)

NB is a simple statistical algorithm based on the Bayes Theorem. It is based on the class conditional independence assumption, which states that the effect of the predictor on the given class is independent of the values of other predictor classes [49]. This assumption makes the algorithm robust and computationally cost-effective to be used in real-time applications. Despite the fact that NB is a simple statistical algorithm, its usability in this study is for the reason that NB does not require a large training data set for the accurate classification [32]. In the context of game player expertise classification, NB is found to be an effective classifier with the 88.89% classification accuracy [41]. In another study, NB classified the expertise of the game player with 84% and 88% classification accuracy when features from 4 and 14 EEG channels were used, respectively [36].

#### K- nearest neighbor (KNN)

KNN is one of the simplest supervised machine learning algorithms widely used in varied problems because of its low computational complexity. The classification process of KNN is relatively simpler as it analyses the feature similarity of each input data point with the output class and classifies the input data point to the output class with which it has the maximum correspondence. It classifies the input data point by finding the distance or closeness of its features from each of the output classes, i.e., expert or novice. The K value in KNN denotes the number of nearest neighbors. The value of *K* chosen in this study is 1. In the context of classifying the game player emotions, KNN is found to be a good classifier. A study conducted by Lin et al. [50] has verified the usability of the KNN classifier in classifying the player experience. Their study’s results have reported that the KNN classifies the four emotions with an accuracy of 82% using the features of the EEG signals. In another study, five different emotions were classified with an accuracy of 82.87%, and 78.57% for the data obtained from 62 and 24 EEG channels when the stimulus used were emotional audio-visual clips [51]. The study conducted by Parsons et al. has identified that the KNN is the best classifier for classifying the general game based events when the beta band is used as a predictor feature [32]. The previous studies in the GUR have used the KNN classifier to classify various player experiences, whereas none has utilized it for the classification of the game player expertise. Nonetheless, the efficiency of the KNN classifier is well known, and because of its optimum performance in the previous studies, it is chosen in the current study for the classification of the game player expertise using the EEG features.

#### Multilayer perceptron (MLP)

MLP is a type of neural network that uses the backpropagation algorithm for classification purposes. This feed-forward neural network-based algorithm works by assigning weights to the input data, which are mapped to each neuron’s output through the transfer function. Thus, a non-linear relationship is established between weighted inputs to the output of the network. The frequently used transfer functions in the MLP classifier are sigmoid, hyperbolic tangent, rectified linear unit, and radial [52]. The transfer function used in this study is the *sigmoid function* and the hidden layers are chosen in *Weka* as parameter *a* i.e. the number of attributes + number of classes / 2. In the context of game player expertise classification, MLP has proved to be a good classifier with the 80% accuracy of expertise classification [41]. In another study, the MLP classifier achieved 82% and 84% classification accuracy when features from 14 and 4 EEG channels were used, respectively [36]. The performance of MLP in the previous studies endorses its use in the current study and future studies.

#### Random forest (RF)

RF is a supervised learning classifier that is widely used because of its robustness, insensitivity to overfitting, and ability to model the categorical values. Its applicability is suited for classification as well as regression tasks. It is an ensemble algorithm that works by creating multiple decision trees by randomly selecting features from the training set and then predicting the test data class by finding out its similarity to all of the decision trees in the forest. RF is an effective classifier for the classification of multiple user states based on their EEG signals while the users are engaged in the game playing [53]. By reviewing the literature, it has been identified that RF is neither utilized for the game player expertise classification nor the classification of player experience. Nonetheless, the applicability and usability of the RF classifier is well known, and therefore, it is used in the current study for the EEG based game player expertise classification.

#### Classification-validation model

In this study, the performance of various classifiers was evaluated using a 10-fold cross-validation (CV) model where all of the trial instances of the game players were divided into 10 splits: 9 splits were used for training the classifier and 1 split for testing the classifier performance. We further used leave-one-out cross validation (LOOCV) and leave-one-subject-out (LOSO) cross validation for evaluating our models. Since the data used in this study was limited, LOOCV was used to verify the classification results along with the 10-fold CV. While LOSO was used to analyze the results for subject-based analysis instead of using all instances independently.

#### Evaluation metrics for the performance of classification

We have used well known performance metrics for analyzing the classifier performance ([36, 41]). In particular, classifier performance of various classifiers was evaluated in terms of classification accuracy and error performance metrics. Moreover, precision, recall, F-measure, and receiver operating characteristic curve (ROC) were also evaluated for each classifier. The performance measures were calculated as follows,

$$\begin{array}{c} Accuracy=\frac{TP+TN}{TP+TN+FP+FN}, \end{array}$$ (4)

$$\begin{array}{c} Precision=\frac{TP}{TP+FP}, \end{array}$$ (5)

$$\begin{array}{c} Recall=\frac{TP}{TP+FN}, \end{array}$$ (6)

$$\begin{array}{c} F-measure=2\times \frac{Precision*Recall}{Precision+Recall}, \end{array}$$ (7)

where true positive, true negative, false positive and false negative observations are denoted by *TP*, *TN*, *FP*, *FN* respectively. ROC represents the plot between the true positive rate (TPR) and the false positive rate (FPR) of the classifier.

## Results

The EEG data obtained from 10 participants were classified into expert and novice categories by thresholding each participant’s average game score (as described in the EEG dataset subsection) followed by the preprocessing step to remove artifacts. The clean EEG signals were then fed to the channel selection step, where each EEG channel’s power was treated as an attribute of interest. Hence, features form EEG channels which were maximally related to the output class were used to classify expertise of game player. The results from each of these stages are presented in the following subsections.

### Performance of channel selection

**Fig 4:**
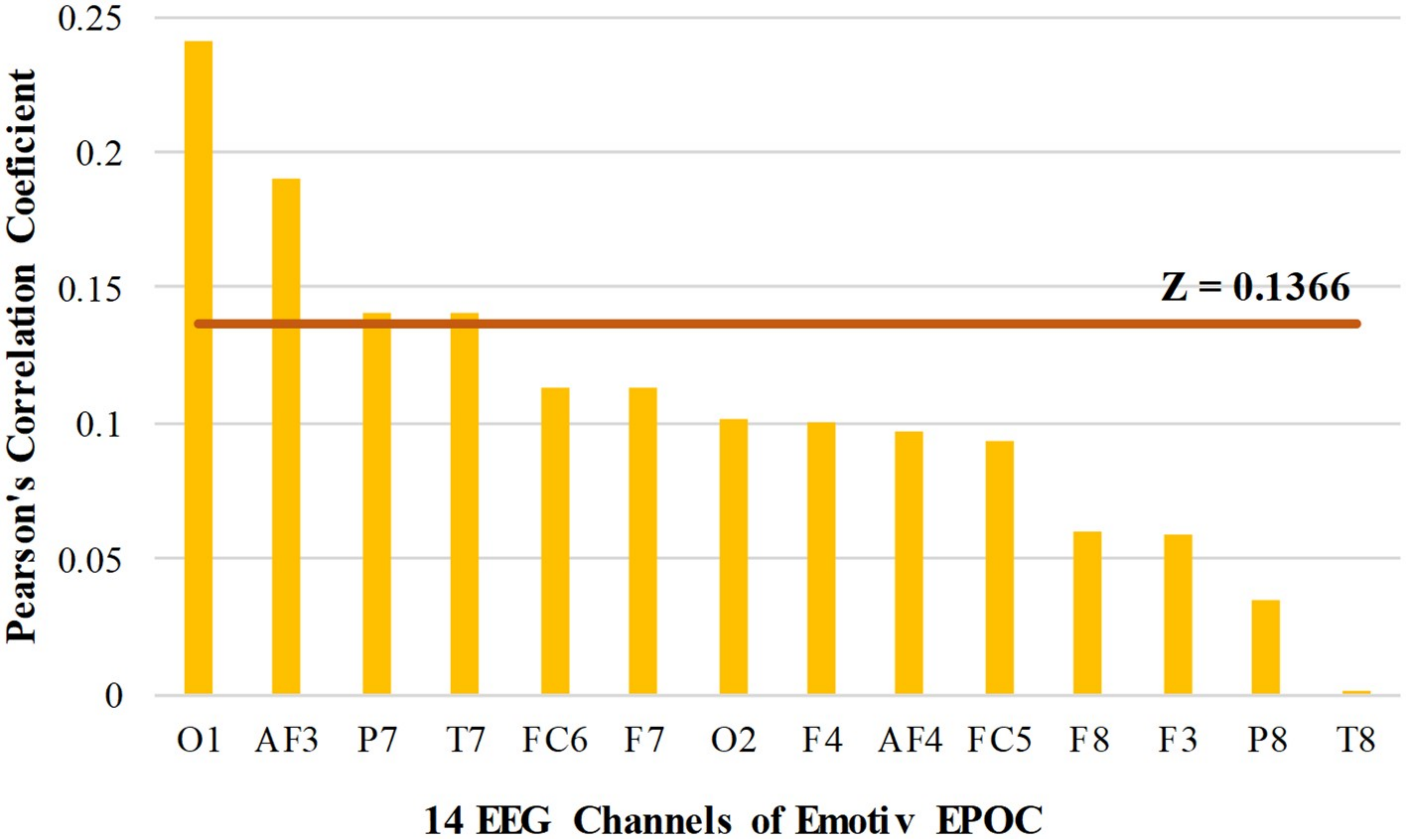
Pearson’s correlation coefficient values of the fourteen channels of Emotiv EPOC obtained by the correlation-based attribute selection method.

Channel selection was performed using *correlation-based attribute selection* method, which evaluated the correlation of the power of each of the fourteen EEG channels with the output class. *Pearson’s correlation coefficient* found for all fourteen EEG channels is shown in Fig 4. The highly correlated EEG channels were chosen using the threshold, $Z=\mu +\frac{\sigma }{2}$, where *μ* and *σ* denote the *mean* and *standard deviation* of the correlation coefficients of the EEG channels. The threshold *Z* gave a value of 0.1366, which suggested that EEG channels O1, AF3, P7, and T7 are highly correlated with the output class compared to the rest of the EEG channels. Channel O1 shows the highest correlation with the output class and has a correlation coefficient value of 0.2408. While AF3, P7, and T7 are ranked in second, third, and fourth-order with correlation coefficient values of 0.1908, 0.1409, and 0.1406, respectively. The rest of the EEG channels, whose correlation value was less than *Z*, were neglected. It is further observed (Fig 4) that correlation values of EEG channels P8 and T8 approached zero, indicating least correlation with the output class.

### Classification performance of the classifiers

Channel selection resulted in identifying four significant channels, namely, O1, AF3, P7, and T7, whose features were used in the feature extraction phase. PSD features of the four significant channels were computed by using the Welch method resulting in five frequency bands. Thus, for four EEG channels, their corresponding five PSD features were computed and arranged in the form of a matrix. A total of 50 trial files were used, consisting of 20 experts and 30 novice instances, and each channel resulted in five PSD features. Thus, 20 features from four significant channels were extracted. Thus, a feature matrix of 50 x 20 dimension was formulated accompanied by the class labels for each instance. The rows of the matrix correspond to the total trial instances of the gameplay, and columns correspond to the PSD features. To get a better insight of which channels contribute most in classifying the game player’s expertise, all combinations of the significant EEG channels O1, AF3, P7, and T7 were computed using the combinatorics formula ${}^{m}C_{r}=C(m,r)=\frac{m!}{r!(m-r)!}$, where *m* is the total number of channels, i.e., four and *r* is the number of channels selected from a total of four channels considering no repetitions of channels. This was done to explore which channel or combination of the channel’s features gives maximum accuracy for classifying the expertise of the game player. Thus, fifteen combinations of the EEG channels (4*C*1 + 4*C*2 + 4*C*3 + 4*C*4) are found, and subsequently, all of their features were analyzed for the classification performance. The features of the selected channels were then fed to the classification algorithms to classify the game player’s expertise. Moreover, this classification procedure led to selecting the best classifier that achieved the maximum classification accuracy.

**Fig 5:**
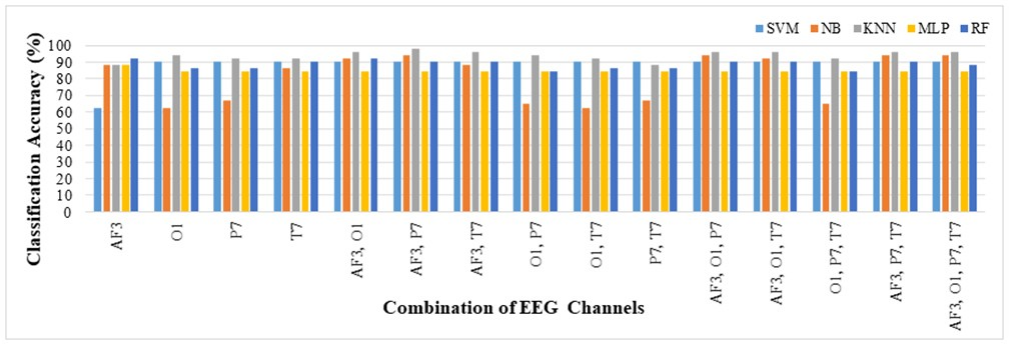
Classification accuracy of the game player’s expertise for fifteen combinations of the EEG channels.

A 10-fold cross-validation model was used for evaluating the classification performance of the features of all fifteen combinations of four EEG channels. Fig 5 depicts the accuracy of game player expertise classification when all fifteen combinations of the significant EEG channels O1, AF3, P7, and T7 were used. The game player’s expertise’s classification results were evaluated for five classifiers SVM, NB, KNN, MLP, and RF. It is observed (Fig 5) that the highest accuracy of 98.04% was achieved when using the KNN classifier with features from AF3 and P7 channels. However, using all features from the selected channels (four) gives the highest classification accuracy of 96.08%, using the KNN classifier. Hence, KNN classifier efficiently classifies the game player expertise using the features of only two significant channels. This indicates that using AF3 and P7 channels only, gives better classification accuracy compared to other channels or their combinations. The improved accuracy of the game player’s expertise classification with less computation achieved by the channel reduction method makes it suitable for the real-time implementation of the DDA based neuro-feedback games.

The performance metrics for all combinations is presented in Table 1, where the least accuracy of 96.08% and F-measure of 0.96 is achieved. These two evaluation metrics show the stability of the classification system. It is worth noting that in all cases, KNN is the best classifier for classifying the expertise of the game player based on accuracy and F-measure, which is the harmonic mean of precision and recall. Moreover, AF3 is a significant EEG channel contributing most to the game player’s expertise classification. This active frontal region’s contribution could be attributed to the fact that game playing involves thought processing, cognition, and decision-making, along with muscle movements [36, 54]. Thus, it is one of the most active regions of the brain that depicts neural activities which contribute most to classifying the game player’s expertise. The parietal brain region is more related to the processing of spatial information. The active processing of this channel is analyzed by the contribution of its features in the classification process.

**Table 1:** Performance comparison of the KNN classifier for the different combination of the EEG channels used.

Channels	Accuracy (%)	Precision	Recall	F-measure	ROC
AF3,P7	98.04	0.98	0.98	0.98	0.98
AF3,O1	96.08	0.96	0.96	0.96	0.97
AF3,T7	96.08	0.96	0.96	0.96	0.95
AF3,O1,P7	96.08	0.96	0.96	0.96	0.97
AF3,O1,T7	96.08	0.96	0.96	0.96	0.97
AF3,P7,T7	96.08	0.96	0.96	0.96	0.95
AF3,O1,P7,T7	96.08	0.96	0.96	0.96	0.97

### Statistical analysis

Statistical analysis (t-test) has been performed on the PSD features of AF3 and P7 channels to confirm the obtained results’ validity. A two-tailed t-test confirmed the significance of these features as all of the features have a p-value less than 0.05. To get a better insight into the brain activity of the two player groups, the distributional characteristics of the AF3 and P7 channels of the expert and novice player groups were analyzed through box plots that identified the differences in various distributional characteristics and frequency bands of the two-player groups. The lower and upper 25% of the data distribution in the box plot represents the lower and upper quartiles, respectively.

**Fig 6:**
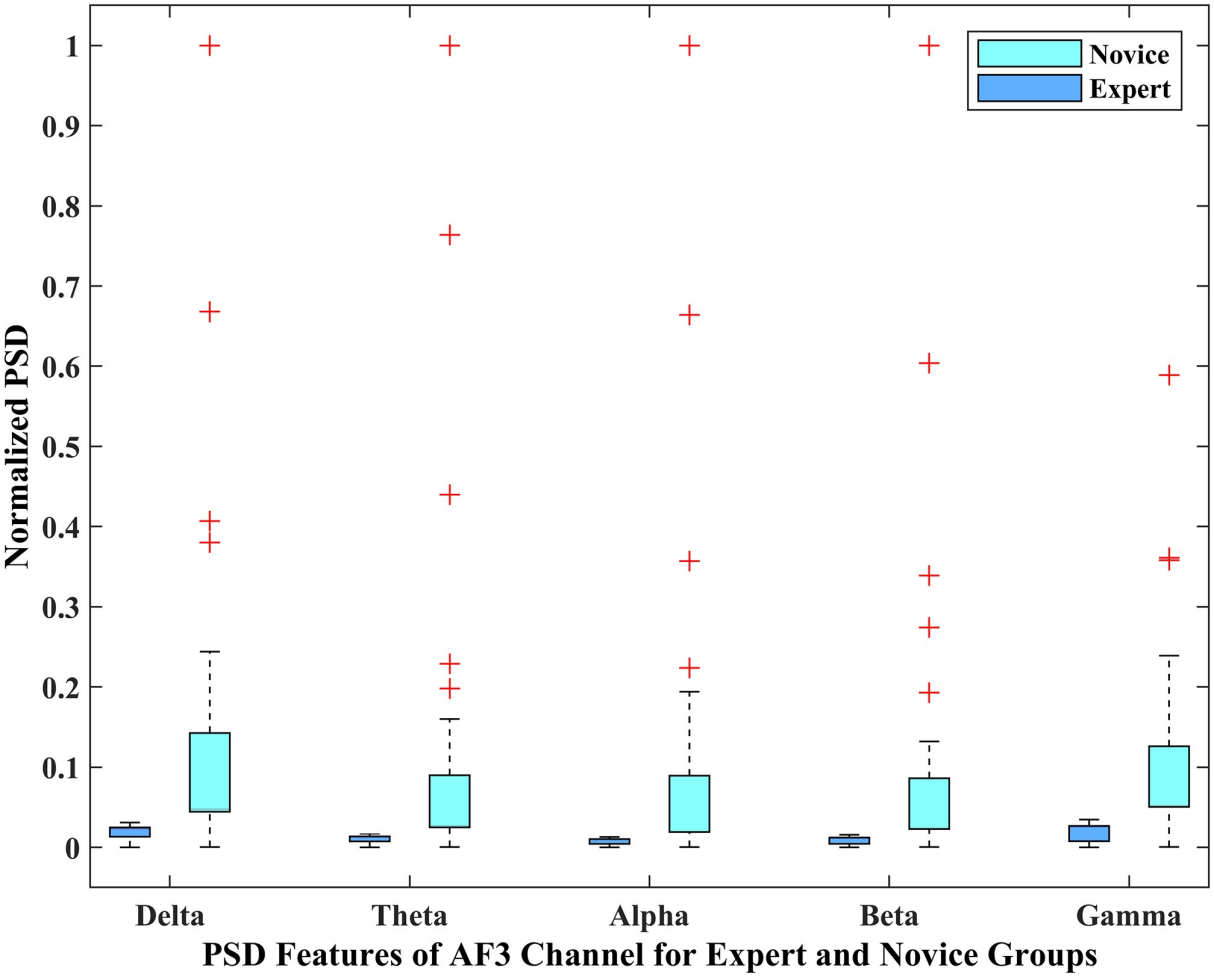
Box-plot representation of the PSD features of *AF*3 channel for expert and novice groups.

**Fig 7:**
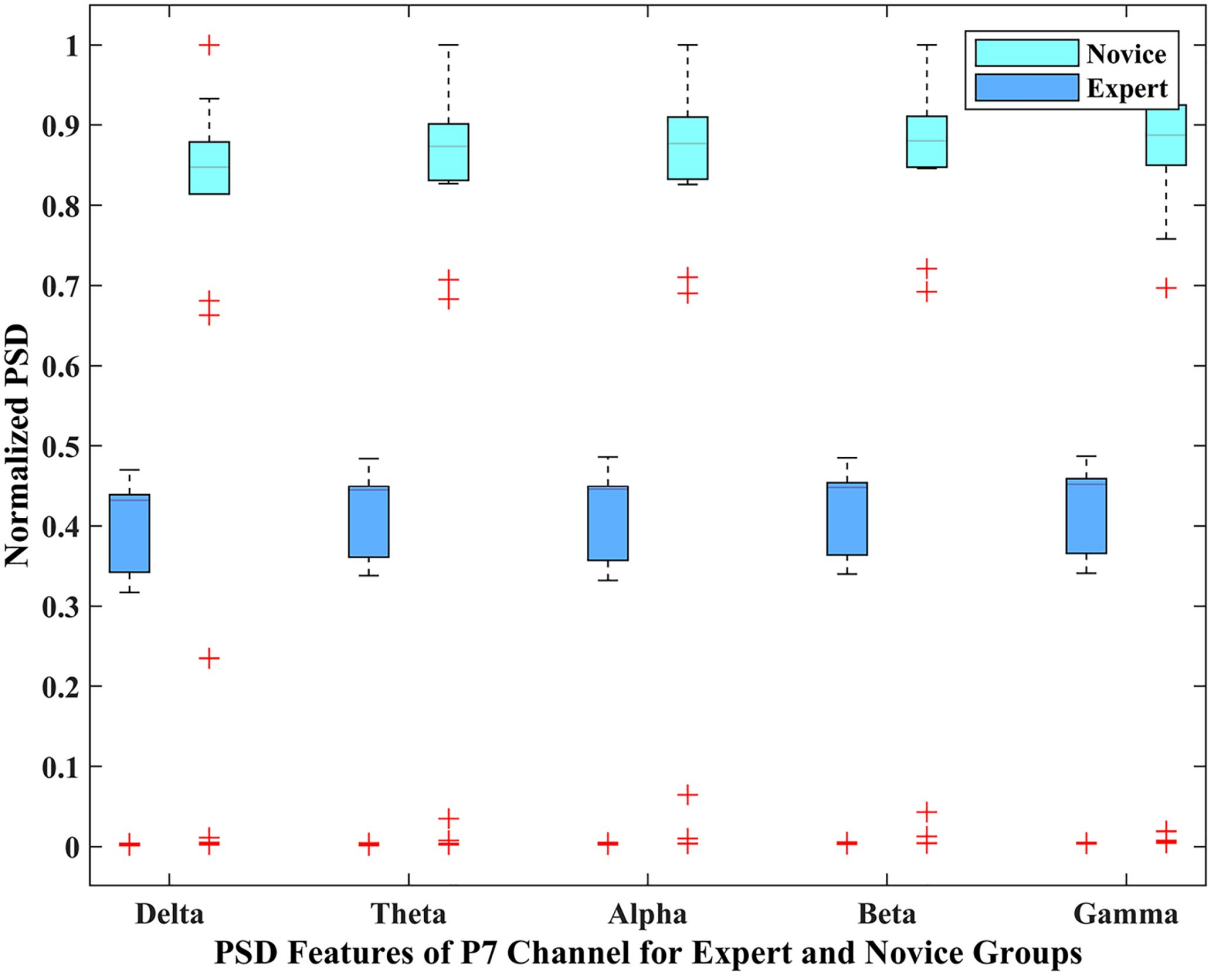
Box-plot representation of the PSD features of *P*7 channel for expert and novice groups.

These are represented by the whiskers, whereas the rest of 50% of the inter-quartile range (IQR) is represented by a box. The horizontal line in the box represents the median of the IQR. The boxplots for PSD features of EEG channel AF3 and P7 are represented in Figs 6 and 7 respectively. The magnitude of PSD is normalized in the range 0 to 1. It is evident that the PSD magnitude of the expert group is visually different from the novice group. Thus, it is obvious from both figures that there is a clear visual distinction for all the EEG bands between the expert and novice groups during the game play, which verifies our hypothesis that the brain activity of the expert group is different from the novice group and supports the findings of the previous studies [35, 36, 41].

## Discussion

This study is aimed at classifying game player’s expertise while optimizing the classification accuracy and reducing the computational overhead. Our results have shown that the proposed scheme achieves an accuracy of 98.33% (with data balancing using SMOTE) by utilizing the features of only two EEG channels using the KNN classifier. The EEG data constitutes six novice and four expert players engaged in five trials of gameplay. Our data were not class-balanced (20 expert and 30 novice trial samples) and therefore could be prone to biased results for the majority class and consequently miss-classifying the minority class [55]. Therefore, SMOTE algorithm [56] was used to synthetically create samples of the minority class thus producing a balanced dataset. The SMOTE algorithm was applied in WEKA with generation of 50% SMOTE instances using 5 nearest neighbors. Consequently, 10 SMOTE instances were created for the expert class, thus producing a balanced data with 30 novice and 30 expert game play samples. The various data configurations used in this study are summarized in Table 2. A 10-fold cross-validation model was used for evaluating the classification performance of the features of all fifteen combinations of four EEG channels. It is observed that, after class-balancing, the highest accuracy of 98.33% was achieved when classified through the KNN classifier using features from the electrodes AF3 and P7.

**Table 2:** The details of the EEG data at each stage of processing.

Description	Raw EEG Dataset	Features from AF3, O1, P7, and T7 EEG Channels (after channel selection)	After Class-balancing (SMOTE)	Classification (features from AF3 and P7 Channels)
No. of Instances	50, Expert = 20, Novices = 30	50, Expert = 20, Novices = 30	60, Expert = 30, Novices = 30	60, Expert = 30, Novices = 30
No. of Classes	2	2	2	2
No. of Channels	14	4	4	2
Feature Matrix Size	–	50x20	60x20	60x10

**Fig 8:**
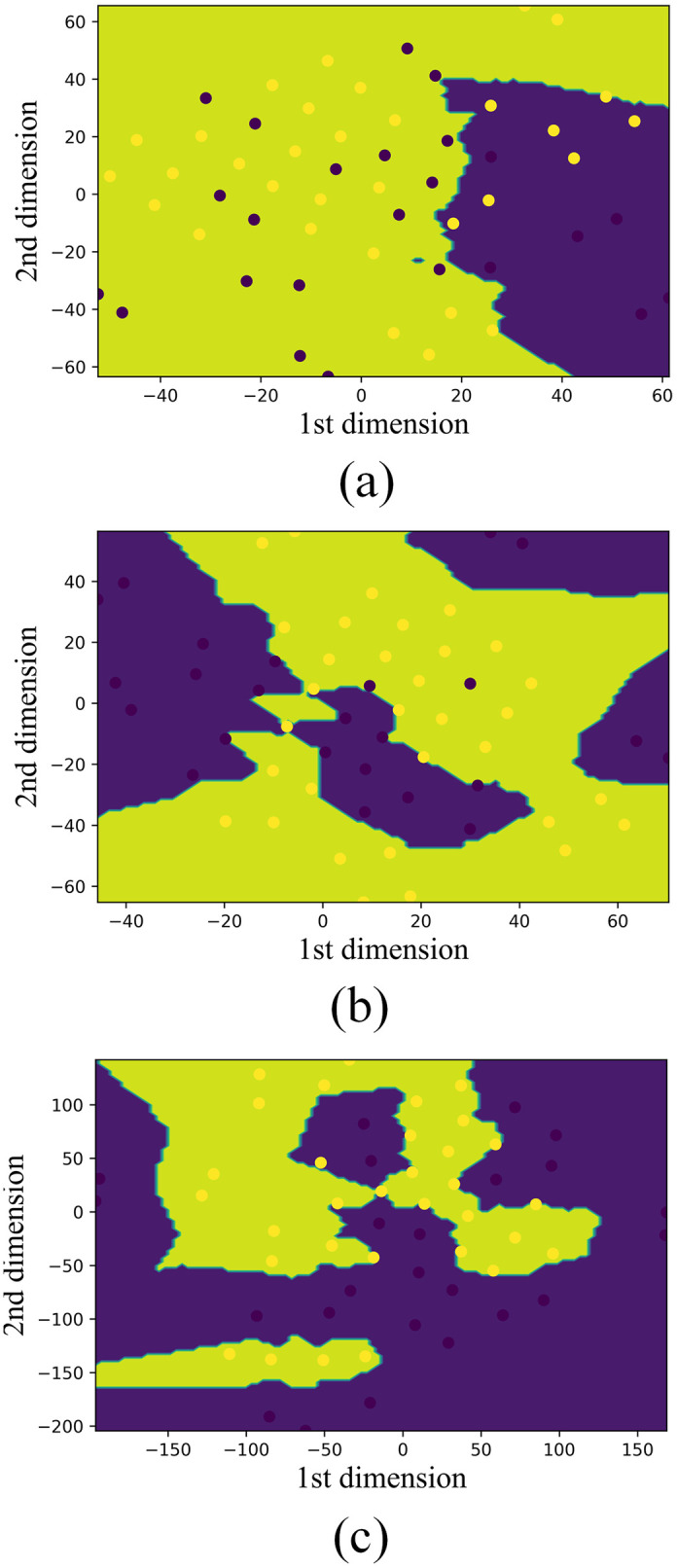
Visualization of features for expert and novice users obtained from (a) all the EEG channels, (b) selected EEG channels, (c) AF3 and P7 channels using t-SNE with decision boundaries.

A visualization of features obtained from all channels as well as selected channels of the Emotiv EPOC EEG headband using t-Distributed Stochastic Neighbor Embedding (t-SNE) and voronoi-based visualization scheme is presented in Fig 8. It is observed that features from all channels resulted in non-discriminating boundary, hence a large number of data points are mis-classified. Whereas, for selected channels (four) the decision boundary becomes more precise resulting in three mis-classified data points (Fig 8(b)). Moreover, using two channels (AF3 and P7), we achieve a more clear decision boundary with only one data point which is mis-classified (Fig 8(c)).

Game player’s expertise was classified using the video game *Guitar Hero*, which requires the attention and coordination to play the musical notes on the musical songs of the rock genre [35]. The study was conducted on eight participants, out of which four were experts, and four were novices. Participants were asked to play two songs in two modes, i.e., easy and difficult. Delta, theta, alpha, and beta band, along with the two ratios of delta/beta and theta/beta, were used as the features for game player expertise classification. Their results showed that expert players’ cognitive activity is different from that of novices, and the accuracy achieved for game player expertise classification was 80%. The study conducted in [41] performed EEG based game player expertise classification using time-domain features and a machine learning approach. Thirteen-time domain features were extracted and classification was performed using SVM, NB, and MLP classifiers. Their results showed that NB classifier best classifies the game player expertise with 89.89% classification accuracy. In another non game-based study [37], expertise classification in the 3D environment using the Normalized Transfer Entropy (NTE) of the EEG features was proposed. Their reported classification accuracy was 95% using five significant features extracted from the EEG data of the frontal electrodes. Comparable to previous studies, our proposed framework of game player expertise classification is based on frequency domain analysis utilizing data from EEG bands. While features from relevant EEG channels were obtained by the Correlation-based feature selection method. The result were analyzed using a 10-fold cross-validation model. Our proposed framework outperforms previous reported methods (Table 3) while utilizing features from two EEG channels (AF3 and P7) and achieved a classification accuracy of up to 98.33% using K-NN classifier. We also performed leave-one-out cross-validation and leave-one-subject-out CV in addition to 10-fold CV. We thereby evaluated the results from the 10-fold CV in terms of accuracy. It should be noted that since the number of training instances was limited, LOOCV was feasible and gave us insights into the classifier performance. The classification accuracy achieved from LOOCV using the KNN classifier was similar to 10-fold CV. While for LOSO, the classification accuracy dropped but was still significant. The performance is bound to increase as more subjects are added. While we observe a drop in performance, but it must be emphasized, multiple trials were performed to augment the data, and we consider each trial instance as an independent subject when performing 10-fold CV. Moreover, the comparison is performed based on the number of electrodes, subjects, stimulus, classification algorithm, and average classification accuracy achieved.

**Table 3:** A comparison of the current study with related studies in game research.

Method	EEG Channels	Dataset (Subjects x Trials)	Stimulus	Classifier	Validation	Accuracy (%)
[35]	14	16 (8x2)	Guitar Hero	Logistic Regression	10-fold	80.00
[41]	14	50 (10x5)	Temple Run	NB	10-fold	89.89
[36]	14	100 (20x5)	Temple Run	NB	10-fold	88.00
[37]	14	8 (8x1)	3-D modelling	KNN	5-fold	95.00
Proposed	2	50 (10x5)	Temple Run	KNN	LOSO	88
Proposed	2	50 (10x5)	Temple Run	KNN	LOOCV	98.04
Proposed	2	50 (10x5)	Temple Run	KNN	10-fold	98.04
Proposed (using SMOTE)	2	60 (12x5—synthetic)	Temple Run	KNN	10-fold (after class-balancing with SMOTE)	98.33

Since, methods used for comparison here are based on different data, for a fairer comparison, we have replicated some of these studies on our data. The results are presented in Table 4, where we observed that the performance evaluated using various evaluation metrics is lower when compared with our proposed method. The proposed methodology has an excellent classification performance using AF3 and P7 electrodes, compared to previous studies and have a value of precision, recall, F-measure and ROC close to 1. AF3 electrode represents the frontal cortex of the brain and P7 belongs to parietal region of the brain. The frontal cortex, along with the other associated cortical areas, performs diverse functions loosely called cognition. In particular, the frontal cortex is especially important for planning appropriate behavioral responses to external and internal stimuli. The frontal cortex integrates complex perceptual information from sensory and motor cortices as well as from the parietal association cortex to perform these cognitive tasks [57] Variability in brain activity of these areas shows that these regions take part in the expertise level of the game player.

**Table 4:** Performance evaluation for various methodologies previously proposed and applied on our data.

	Features	Accuracy (%)	Performance Evaluation			
			Precision	Recall	F-measure	ROC
[35]	FD	LR: 72.55	0.733	0.725	0.728	0.747
[41]	Thirteen TD	SVM: 70.59	0.697	0.706	0.694	0.65
		NB: 72.55	0.733	0.725	0.728	0.801
		MLP: 68.63	0.681	0.686	0.682	0.747
[36]	Thirteen TD	SVM: 72.55	0.726	0.725	0.704	0.664
		NB: 68.63	0.686	0.686	0.686	0.742
		MLP: 74.51	0.741	0.745	0.735	0.742
Proposed (using SMOTE)	FD	K-NN: 98.33	0.984	0.983	0.983	0.983

FD: frequency domain, TD: time domain.

Despite obtaining the highest accuracy of classification, the results of our analysis should be considered with some limitations to understand the scope and applicability of our research. Nonetheless, a larger sample of data is needed to expand the current findings and develop methodological procedures to classify the expertise of the game player in the various game genre. The shortcomings of the current study should be further validated through experiments utilizing multiple game stimuli. Moreover, the effect of movement, environment, and system parameters can also be incorporated to see the effect on the behavior of expert-novice gameplay. Moreover, while a high accuracy is achieved, such experimental protocols have to be carefully designed for clinical application. In particular, the accuracy of wearable EEG headsets needs careful attention when dealing with more critical application areas. The findings of the current study will be beneficial for the development of adaptive games that adjust themselves as per the physiological changes occurring in the game player. Furthermore, the brain activity of the different game player groups can be supplemented in the game design (as in the neurofeedback games), which will gauge the player to preserve his interest and engagement. Such kind of adaptive game designs are found to be more engaging and help the game players to achieve the desired skill- competence balance as well as improve the attention and cognition of the game player [22, 28]. In the future, we intend to overcome the limitations of the current study by increasing the sample size and utilizing a diverse set of games. At the same time, the analysis presented here has emphasized the applicability and usability of EEG signals acquired using wearable devices, such as Emotiv EPOC headset, towards evaluating game player expertise. Our experimental results are found to be significant, and hence, in the future, we intend to evaluate various modalities of game player experience.

## Conclusion

This study presents EEG based game player expertise classification using an objective, reliable, and discreet method which is less prone to subjective or biased opinions. Moreover, while working with the EEG signals, it is desirable to reduce the computational overhead. Our experimental results demonstrated that the brain activity of expert and novice game players has noticeable differences, and the expertise of game players is classified with 98.33% accuracy using the KNN classifier. While this is achieved by utilizing features from two EEG channels: AF3 and P7. These findings could be used in the development of DDA based neuro-feedback games, which adapt their game design in correspondence with the changes occurring in the physiological and electrophysiological signals of the game player. Further, digital interfaces, including video and mobile games, can be made more engaging and interactive by incorporating the player’s expertise and experience as a part of the game design. Thus, our results emphasize the efficacy of analyzing cognitive and behavioral aspects of game players to design games that keep the player engaged and motivated during the gameplay. In future, we intend to predict the score of a game player using EEG signals and regression models. Furthermore, we shall collect more data from subjects engaging with a diverse set of mobile games for an in-depth analysis of game users, which would allow us to apply deep learning for both classification and feature extraction. Our aim in this direction is to use explainable deep learning methods that can work on small amount of training data. Moreover, the engagement behavior of the game player will be investigated using EEG based engagement indices.
